# Analysis of Pharmaceutical Industry Payments to UK Health Care Organizations in 2015

**DOI:** 10.1001/jamanetworkopen.2019.6253

**Published:** 2019-06-21

**Authors:** Piotr Ozieranski, Marcell Csanadi, Emily Rickard, Jordan Tchilingirian, Shai Mulinari

**Affiliations:** 1 Department of Social and Policy Sciences, University of Bath, Bath, United Kingdom; 2 Syreon Research Institute, Budapest, Hungary; 3 Department of Sociology, Lund University, Lund, Sweden

## Abstract

**Importance:**

Drug company payments to health care organizations can create conflicts of interest. However, little is known about such financial relationships, especially outside the United States.

**Objective:**

To examine the concentration and patterns of drug company payments to health care organizations in the United Kingdom.

**Design, Setting, and Participants:**

This cross-sectional study examined nonresearch payments reported in the industry-run Disclosure UK database. Companies participating in Disclosure UK in 2015 and health care organizations receiving their payments were included in the analysis. The data were analyzed descriptively at the health care organization, payment, and donor levels, considering health care organization categories, payment categories, and companies from February 5 through May 28, 2017, with follow-up checks from June 1 through August 31, 2018. Analysis was conducted from July 10 through December 20, 2018.

**Main Outcomes and Measures:**

Share of funding and the Gini index (GI) to measure payment concentration (0 indicates perfect deconcentration [eg, all drug companies provide the same value of payments]; 1, perfect concentration [eg, 1 company provides the entire value of payments]) and median and interquartile range (IQR) to measure payment patterns.

**Results:**

A total of 4028 health care organizations received 19 933 payments, worth US $72 110 156.6, from 100 companies. This study identified 11 categories of health care organizations, with 3—public-sector secondary and tertiary care providers, education and research providers, and professional organizations—accumulating 67.2% of funding. The health care organization categories had varying GIs (range, 0.65-0.92), medians (range, $750.3-$45 862.4), and IQRs (range, $389.1-$1843.9 to $3104.4-$199 868.2). Of 4 payment categories, the top category—donations and grants—captured 50.6% of funding. Joint working (collaborative projects with nonindustry partners) had a lower GI (0.64) than other payment categories (range, 0.79-0.84). The median and IQR were the lowest for contributions to costs of events ($366.8; IQR, $229.3-611.3) and highest for joint working ($14 903.7; IQR, $3185.0-34,748.4). The top 10 firms (58.6% of funding) had payments with varying medians (from $366.8 [IQR, $244.5-611.3] to $9781.3 [IQR, $1834.0-48 906.7]).

**Conclusions and Relevance:**

Although organizations from across the health care system received funding, the payments were concentrated on a few large donors, payments, and recipients. Different payment and recipient categories had different patterns of payment values, suggesting that the industry has diversified its funding strategies across different parts of the health care system. These results suggest that Disclosure UK requires improved transparency, particularly by including built-in recipient categories, and that organizational conflicts of interest need more policy attention, including disclosure of payments independent of the industry.

## Key Points

**Question:**

How are drug company payments to health care organizations distributed in the UK health care system?

**Findings:**

This cross-sectional study of the Disclosure UK database found that in 2015, 4028 health care organizations received US $72 110 156.6 from 100 companies. Although financial relationships were spread across the health care system, a few key donors and beneficiaries of industry funding were found.

**Meaning:**

More policy attention is needed to disclose organizational conflicts of interests, particularly in areas of the health care system with a high concentration of industry payments.

## Teaser

This cross-sectional analysis of the Disclosure UK database examines the concentration and patterns of drug company payments to health care organizations in the United Kingdom.

## Introduction

Unlike in the United States,^1,2,3,4^ little European research on pharmaceutical industry payment disclosures has been performed. This lack is unsurprising given the pervasive nondisclosure of payments by health care professionals in many countries.^5,6^ The ability to refuse to disclose received payments results from the interpretation of European privacy law by individual companies and national pharmaceutical industry trade groups managing the disclosure process in most countries.^7^ These privacy provisions do not extend to health care organizations (HCOs),^8^ but research is impractical because the European self-regulatory disclosure system does not require the establishment of centralized payment databases adaptable for efficient analysis.^7^

The only exception is Disclosure UK, a freely accessible database maintained by the Association of the British Pharmaceutical Industry (ABPI), representing firms that provide the National Health Service (NHS), the United Kingdom’s single-payer health system, with more than 80% of patented drugs according to their value.^9^ All ABPI members must disclose their payments.^10^ In 2015, the first year Disclosure UK operated, 52 of 53 ABPI members reported payments to HCOs, and 49 other drug companies did so voluntarily.^11,12^

The ABPI rules allow payments to HCOs as part of marketing activity, provided that company involvement is made clear to audiences; payments are not an inducement to prescribe, supply, administer, recommend, buy, or sell any medicine; and payments are reported in Disclosure UK.^10^ The payment categories in Disclosure UK are contributions to costs of events; donations and grants; fees for service and consultancy; and joint working (payments associated with collaborative projects with nonindustry partners) (eBox 1 in the Supplement).^13^ Unlike in the United States, individuals and organizations receiving payments for research and development are not disclosed, precluding granular analysis of research payments, and ownership or similar interests are excluded entirely. Another difference is that although the only HCO type included in the Open Payments program is teaching hospitals,^14,15^ Disclosure UK covers many HCO types, including health care providers and professional organizations, such as hospitals, clinics, foundations, universities or other teaching institutes, and learned societies (eBox 2 in the Supplement).^10^

The complexity of the United Kingdom’s single-payer system^16^ allows us to study industry relationships with diverse HCOs. Government funding ($3130.1 per capita and 79.4% of overall health expenditure as of 2016^17^) is primarily channeled through the NHS,^18^ especially its regulatory organizations (which set standards for health care delivery), commissioning organizations (which procure health services), primary care providers (medical practices), secondary care providers (hospitals delivering planned or emergency care), and tertiary care providers (hospitals offering specialist treatment).^19^ The providers of government-funded care can be publicly owned (NHS hospitals), private sector (medical insurance companies), or third sector (charities).^19^ The total spending on non-NHS organizations, including local authorities and private- and third-sector organizations, amounts to 10.9%.^20^

The United Kingdom prioritizes collaboration between the government-funded health sector and the pharmaceutical industry.^21,22^ Although official NHS guidelines recognize that organizational conflicts of interest may arise,^23^ they are not always effectively disclosed by commissioners^24^ and hospitals.^25^ These concerns reflect those identified elsewhere that industry-HCO financial relationships may jeopardize HCOs’ independence,^26,27,28,29^ for example, by biasing HCOs to alter their internal operation to facilitate the industry’s commercial or policy goals.^30^

This study is, to our knowledge, one of the first attempts at building a national-level picture of these ties.^15,31^ We examined the concentration of payments to identify the extent to which payment distributions were dominated by a few major donors, recipients, and payments. We analyzed patterns in payment values, namely central tendency and spread, to show how much payments varied across payment categories, HCO categories, and drug companies.

## Methods

### Data Source

We analyzed Disclosure UK^11^ coverage of nonresearch payments, reported on a per activity basis, made in 2015.^10^ This report follows the Strengthening the Reporting of Observational Studies in Epidemiology (STROBE) reporting guideline. The ethical implications of the study presented in this article were reviewed and approved via a peer ethics review process at the Department of Social and Policy Sciences, University of Bath, Bath, United Kingdom, in April 2016. The Social Sciences Research Ethics Committee at the University of Bath confirmed in April 2019 that a full ethical approval was not required because the data were publicly available as well as analyzed and reported at the organizational level.

### Categories of HCOs

Because Disclosure UK does not include HCO categories, we created them for the purposes of this study. In comparing the funding recipients, we focused on the function HCOs had within the health care system (eg, service provider or professional organization) and their sector (eg, public or private), with specific (mutually exclusive) categories emerging inductively through iterative reading of online descriptions of HCOs. Our categorization (eTable 1 in the Supplement) includes 3 levels of detail, but herein we refer to the most general one.

In categorizing HCOs, we googled the recipient of each payment, refining ambiguous searches by adding recipient postal codes reported in Disclosure UK. We categorized recipients as unclear if their names were missing; if names were stated as geographical locations (eg, building names), individuals, or more than 1 organization; if located outside the United Kingdom; or if the same postal code was associated with more than 1 organization. Two of us (P.O. and E.R.) conducted the web searches and categorization from February 5 through May 28, 2017, and follow-up checks from June 1 through August 31, 2018, with intercoder reliability ensured throughout.

### Naming HCOs

Disclosure UK identifies HCOs using their names and locations, with the latter being separate from addresses. Their meaning is not specified, but companies must provide names, whereas locations are optional.^10,13^ The entries in names and locations differed in relation to 16 335 of 20 040 payments (81.5%), typically pointing to different organizational characteristics of the same recipients. For example, names referred to an NHS hospital, whereas locations referred to the NHS trust, a higher-level organizational unit, to which the hospital belonged.^32^

Given these differences, we applied the same categorization to names and locations separately. We report our results by HCO names and categories based on locations unless they were categorized as unclear; in that case, they were based on names. We took this approach given the better quality of data reported in locations (eMethods in the Supplement). The HCO categories in names and locations disagreed in relation to 2093 of 20 040 payments (10.4%). These payments were spread across different donors and HCO and payment categories. We kept them in the analysis because the general rule of prioritizing information from locations allowed for resolving these discrepancies (eMethods in the Supplement). In creating the list of HCO names reported herein, we addressed inconsistencies in the naming of HCOs in Disclosure UK, including the same HCO being referred to with different names and different HCOs appearing under the same name (eMethods in the Supplement).

### Exchange Rate and Adjustment for Value-Added Tax

We converted payment values from pounds sterling to US dollars using the 2015 annual average exchange rate of £1 = $1.53.^33^ Disclosure UK allows companies to choose how they report value-added tax (VAT) associated with their payments. Therefore, to compare payment values reliably,^34^ we considered company approaches to VAT reporting. We extracted the VAT approaches from methodological notes^10,34^ describing how companies reported their payments. We subtracted 20% (the United Kingdom’s main VAT business rate) from the value of payments by companies reporting gross payments (n = 35), with no single rule on VAT (n = 27), or providing no VAT policy (n = 6) (eTable 2 in the Supplement).

### Statistical Analysis

We analyzed the data from July 10 through December 20, 2018. We used Excel (Microsoft Corp) to analyze the concentration of payments using Gini indexes (GIs) and the shares of the value of payments held by the top 10% and bottom 75%. The GI typically measures inequality in income or wealth distribution at the level of individual recipients.^35^ We applied the GI to examine the concentration of payments at the recipient, payment, and donor levels. The GI considers all observations and compresses the relative difference between individual payments provided or received into a single figure, with values ranging from 0 (perfect deconcentration [eg, all drug companies provide the same value of payments]) to 1 (perfect concentration [eg, 1 company provides the entire value of payments]). We illustrate the GIs with the Lorenz curve (eFigures 1-3 in the Supplement), showing the share of the value of funding (y-axis) cumulatively received by, associated with, or provided by the bottom *x*% of HCOs, payments, or donors, respectively. We compare the Lorenz curve with the 45° line denoting the hypothetical equal distribution of payments. We analyzed payment patterns using the median and interquartile range (IQR), given the lack of normal distribution and large differences between minimum and maximum values.^5^

## Results

Disclosure UK included 20 040 nonresearch payments worth $86 595 160.1 made by 102 companies to 4069 HCOs in 2015. We could not analyze 595 payments (2.9%) worth $3 228 265.5 (3.7%) that 19 companies (18.6%) made to undisclosed HCOs (in apparent violation of ABPI rules^8^). We excluded 107 payments (0.5%) worth $1 617 216.9 (1.9%) that 27 companies (26.5%) made to individuals, organizations based outside the United Kingdom or in Crown Dependencies, more than 1 organization, to sponsor events without HCO names mentioned, and those made by Sigma-Tau (acquired by Baxalta, who reported the same payments).^36^ We also excluded Mundipharma because all its payments fell into the excluded categories.

Overall, we analyzed 19 933 payments that 100 companies made to 4028 UK HCOs. The funding value, after adjusting for company VAT approaches, was $72 110 156.6 (down from $84 977 943.0 before the adjustment). These data included 158 payments (0.8%) worth $339 623.3 (0.5%) to 89 unclear recipients (2.2%).

### Health Care Organizations

Industry funding concentrated on a relatively small number of HCOs. Calculated using the amount of funding per HCO, the GI was 0.89 (eFigure 1 in the Supplement). Correspondingly, the top 10% of HCOs accumulated 86.1% of funding, with the 10 largest recipients (0.002%) alone amassing 19.4% (eTable 3 in the Supplement). The bottom 75% received 5.2%.

Eleven categories of HCOs from across the health care system received funding (Table 1). However, the top 3—public-sector secondary and tertiary care providers, education and research providers, and professional organizations—received 67.2% of funding (Table 2). Overall, regarding the sector of the HCOs (Table 1), public-sector organizations were the main funding target (4 categories, 41.7% of funding, 66.5% of HCOs), followed by third-sector organizations (3 categories, 22.6% of funding, 17.5% of HCOs). Separately, regarding the function of the HCOs, health care provider organizations were predominant (5 categories, 38.4% of funding, 65.1% of HCOs), followed by professional organizations (1 category, 17.3% of funding, 11.8% of HCOs) (Table 1).

**Table 1.** Examples of HCO Categories

HCO Category Name and Function	HCO Category Sector	Examples
Alternative providers of health services	Third	Charities, not-for-profit companies, social enterprises, and community interest companies providing health services
Education and research providers	Mixed (public and third)	Universities, charities, and noncommercial institutes undertaking research
Formal bodies representing health care professionals or patients	Mixed (public and third)	Local medical, optical, optometric, or pharmaceutical committees and statutory bodies representing patients
Organizations supporting patients, health care professionals, or other organizations	Third	Organizations focusing on supporting education, research, advocacy, and multipurpose organizations
Private companies other than providers of health services	Private	Providers of medical communications or training services, commercial or medical research services, and accountancy or consulting services
Private-sector health care providers	Private	Private clinics and hospitals, health care groups, and providers of dental, pharmacy, and optical services
Professional organizations	Third	Organizations of medical professionals or other health care professionals and other professional organizations (non–health care professionals)
Public administration and providers of public services	Public	Central UK government bodies, devolved administrations in Scotland, Wales, and Northern Ireland, and local authorities
Public-sector health care commissioning, planning, and regulatory organizations	Public	Local, regional, and commissioning, planning, or regulatory organizations
Public-sector primary care providers	Public	General practitioner surgeries, medical practice health centers, groups of surgeries or medical practices, and health care or medical groups
Public-sector secondary and tertiary care providers	Public	NHS trusts, NHS hospitals, and networks and collaboratives of NHS organizations

Abbreviations: HCO, health care organization; NHS, National Health Service.

**Table 2.** Categories of HCOs^a^

Level of Analysis, Measure	Category										
	Alternative Providers of Health Services	Education and Research Providers	Formal Bodies Representing Health Care Professionals or Patients	Organizations Supporting Patients, Health Care Professionals, or Other Organizations	Private Companies Other Than Providers of Health Services	Private Sector Health Care Providers	Professional Organizations	Public Administration and Providers of Public Services	Public Sector Health Care Commissioning, Planning, and Regulatory Organizations	Public Sector	
										Primary Care Providers	Secondary and Tertiary Care Providers
HCO level and payment level, total value, $ (%)	152 550.5 (0.2)	15 245 966.9 (21.1)	196 531.2 (0.3)	3 689 209.7 (5.1)	7 614 059.0 (10.6)	2 363 319.4 (3.3)	12 467 577.6 (17.3)	192 623.9 (0.3)	4 672 118.7 (6.5)	4 412 970.1 (6.1)	20 763 606.4 (28.8)
HCOs, No. (%)	68 (1.7)	70 (1.7)	77 (1.9)	161 (4.0)	265 (6.6)	141 (3.5)	477 (11.8)	24 (0.6)	242 (6.0)	2134 (53.0)	280 (7.0)
Payment level, No. (%)	189 (0.9)	1063 (5.3)	489 (2.5)	686 (3.4)	1533 (7.7)	573 (2.9)	2189 (11.0)	30 (0.2)	3312 (16.6)	2909 (14.6)	6802 (34.1)
HCO level, payment, median (IQR), $	750.3 (305.7- 2055.3)	45 862.4 (3104.4- 199 868.2)	886.4 (389.1- 1843.9)	6113.3 (1092.8-24 461.7)	3166.2 (1012.5- 14 794.3)	831.4 (317.9-3285.9)	2751.0 (897.4-14 524.8)	801.8 (339.9-2696.0)	2967.1 (930.3-8623.7)	855.9 (332.0-2139.7)	17 128.5 (3660.4- 75 640.2)
Payment level, payment, median (IQR), $	305.7 (244.5-658.1)	1528.3 (509.4-6113.3)	305.7 (229.3-385.1)	489.1 (275.1-3631.3)	458.5 (300.8-1146.3)	382.1 (244.5-1222.7)	733.6 (367.2-3668.0)	649.5 (330.1-1146.3)	336.2 (227.4-547.8)	664.1 (305.7-1379.3)	366.8 (220.1-764.2)
HCO level, top 10% share, %	56.5	60.6	60.9	56.5	78.1	88.7	72.4	87.5	77.5	47.9	58.9
Payment level, top 10% share, %	56.6	78.6	32.9	72.8	84.8	85.1	69.4	89.1	77.4	50.5	83.9
HCO level, bottom 75% share, %	21.2	13.9	22.4	17.3	8.2	3.5	8.4	6.2	9.7	24.8	15.6
Payment level, bottom 75% share, %	27.4	7.3	49.9	8.0	6.0	6.6	10.9	6.0	14.8	25.2	7.5
HCO level, Gini index^b^	0.68	0.78	0.68	0.73	0.85	0.92	0.82	0.88	0.84	0.65	0.88
Payment level, Gini index^b^	0.63	0.86	0.38	0.83	0.88	0.87	0.80	0.89	0.79	0.64	0.86

Abbreviations: HCO, health care organization; IQR, interquartile range.

^a^ Payment values are expressed in US dollars. The 2015 annual average exchange rate from pounds sterling was used for the conversion (£1 = $1.53).

^b^ Zero indicates perfect deconcentration (eg, all drug companies provide the same value of payments); 1, perfect concentration (eg, 1 company provides the entire value of payments).

In 8 HCO categories, large discrepancies occurred between the shares of HCOs and funding they received. In particular, considerably higher funding shares relative to their HCO numbers were noted for education and research providers (21.1% relative to 1.7%) and for public-sector secondary and tertiary care providers (28.8% relative to 7.0%). The opposite was true for public-sector primary care providers (6.1% relative to 53.0%) and alternative health providers (0.2% relative to 1.7%).

Using the funding value per HCO, public-sector primary care providers had the lowest concentration (GI = 0.65), and private-sector health care providers had the highest concentration (GI = 0.92), with the remaining HCO categories being spread between the 2 extremes. We found no pattern that a higher number of HCOs would indicate lower concentration or vice versa. The hierarchy of concentration was similar at the level of individual payments, with the corresponding GIs and top 10% and bottom 75% shares.

Different HCO categories had different patterns of payment values. The median values of funding aggregated at the HCO level ranged from $750.3 (IQR, $305.7-$2055.3) for alternative health care providers to $45 862.4 (IQR, $3104.4-$199 868.2) for education and research providers. The IQRs ranged from $389.1 to $1843.9 (median, $886.4) for formal bodies representing health care professionals or patients to $3104.4 to $199 868.2 (median, $45 862.4) for education and research providers.

The order of medians and IQRs was broadly similar at the HCO level and the level of individual payments, with 2 notable exceptions. Public-sector secondary and tertiary care providers had a top place at the HCO level (median, $17 128.5; IQR, $3660.4-$75 640.2) and a low one at the payment level (median, $366.8; IQR, $220.1-$764.2), suggesting that the large number of payments (6802 [34.1%]) compensated for their relatively low value because they were provided to a small number of HCOs (280 [7.0%]). Conversely, public-sector primary care providers had a relatively smaller number of payments (2909 [14.6%]) of higher value (median, $664.1; IQR, $305.7-$1379.3) that were dispersed across more HCOs (2134 [53.0%]), translating into lower values at the HCO level (median, $855.9; IQR, $332.0-$2139.7).

### Payments

Industry funding was also concentrated at the payment level. The GI for all payments was 0.85 (eFigure 2 in the Supplement), and the corresponding shares of the top 10% and bottom 75% payments were 81.8% and 7.9% of funding. The top payment category consisted of donations and grants (50.6% of funding) (Table 3). Gini indexes were similar for contributions to costs of events (0.79), donation and grants (0.83), and fees for service and consultancy (0.84). The corresponding shares of the top 10% were worth 76.2% of the total for contributions to costs of events, 74.3% of the total for donations and grants, and 78.0% of the total for fees for service and consultancy. Corresponding shares of the bottom 75% of payments were worth 9.8% of the total for donations and grants and for fees for service and consultancy and 14.7% of the total for contribution to costs of events. Joint working had a lower concentration (GI = 0.64), with the top 10% and bottom 75% of payments constituting 48.3% and 27.9%, respectively, of the total.

**Table 3.** Payment Categories^a^

Measure	Contribution to Costs of Events	Donations and Grants	Fee for Service and Consultancy	Joint Working
Total value, $ (%)	21 447 638.3 (29.7)	36 487 990.0 (50.6)	10 027 297.0 (13.9)	4 147 230.6 (5.8)
No. (%) of payments	13 964 (70.1)	4425 (22.2)	1406 (7.1)	138 (0.7)
Payment, median (IQR), $	366.8 (229.3-611.3)	1108.2 (332.0-4615.6)	1146.3 (397.4-3056.7)	14 903.7 (3185.0-34 748.4)
Top 10% share, %	76.2	74.3	78.0	48.3
Bottom 75% share, %	14.7	9.8	9.8	27.9
Gini index^b^	0.79	0.83	0.84	0.64

Abbreviation: IQR, interquartile range.

^a^ Payment values are expressed in US dollars. The 2015 annual average exchange rate from pounds sterling was used for the conversion (£1 = $1.53).

^b^ Zero indicates perfect deconcentration (eg, all drug companies provide the same value of payments); 1, perfect concentration (eg, 1 company provides the entire value of payments).

As with HCO categories, different payment categories had varying value patterns. Contributions to costs of events had the lowest median ($366.8) and IQR ($229.3–$611.3), whereas joint working had the highest median ($14 903.7) and IQR ($3185.0-$34 748.4) (Table 3). The medians of donations and grants ($1108.2) and fees for service and consultancy ($1146.3) were similar and situated between the 2 extremes. Donations and grants, however, had a higher IQR ($332.0-$4615.6) than fees for service and consultancy ($397.4-$3056.7).

Certain payment categories concentrated on certain HCO categories (Table 4). Contributions to costs of events constituted more than 50% of funding received by formal bodies representing health care professionals or patients (94.2%), professional organizations (67.6%), and alternative providers of health services (56.0%). Fees for service and consultancy constituted 76.1% of funding received by private-sector health care providers; and donations and grants constituted more than 50% of funding received by public administration (92.1%), public-sector primary (83.0%) and secondary (59.2%) care providers, organizations supporting patients, health care professionals, or other organizations (60.8%), and education and research providers (56.5%). The highest value of funding associated with joint working among all HCO categories was 20.0% in the case of health care commissioning, planning, and regulatory organizations.

**Table 4.** Categories of HCOs and Payments^a^

Measure	HCO Category, Total Payment, $ (%)										
	Alternative Providers of Health Services	Education and Research Providers	Formal Bodies Representing Health Care Professionals or Patients	Organizations Supporting Patients, Health Care Professionals, or Other Organizations	Private Companies Other Than Providers of Health Services	Private Sector Health Care Providers	Professional Organizations	Public Administration and Providers of Public Services	Public Sector		
									Health Care Commissioning, Planning, and Regulatory Organizations	Primary Care Providers	Secondary and Tertiary Care Providers
Contribution to costs of events	85 384.5 (56.0)	2 238 586.1 (14.7)	185 202.3 (94.2)	1 199 098.9 (32.5)	2 024 839.1 (26.6)	330 776.1 (14.0)	8 426 852.5 (67.6)	14 499.6 (7.5)	1 633 574.5 (35.0)	683 113.5 (15.5)	4 460 139.9 (21.5)
Donations and grant	61 054.3 (40.0)	8 607 582.6 (56.5)	1880.5 (1.0)	2 243 205.5 (60.8)	3 461 979.6 (45.5)	192 676.3 (8.2)	3 704 974.0 (29.7)	177 360.1 (92.1)	1 920 848.7 (41.1)	3 660 809.1 (83.0)	12 301 927.0 (59.2)
Fee for service and consultancy	6111.7 (4.0)	4 080 376.2 (26.8)	9448.6 (4.8)	224 750.5 (6.1)	2 075 062.8 (27.3)	1 797 859.4 (76.1)	324 441.6 (2.6)	764.2 (0.4)	182 041.2 (3.9)	69 047.5 (1.6)	1 237 033.8 (6.0)
Joint working	0.0 (0.0)	319 421.8 (2.1)	0.0 (0.0)	22 154.7 (0.6)	52 177.3 (0.7)	42 007.8 (1.8)	11 309.7 (0.1)	0.0 (0.0)	935 654.3 (20.0)	0.0 (0.0)	2 764 505.7 (13.3)

Abbreviations: HCO, health care organization.

^a^ Payment values are expressed in US dollars. The 2015 annual average exchange rate from pounds sterling was used for the conversion (£1 = $1.53).

### Drug Companies

Funding was similarly concentrated at the donor level (GI = 0.74) (eFigure 3 in the Supplement), with the corresponding shares of the top 10 and bottom 75 donors being 58.6% and 16.6%, respectively. Among the top 10 companies, the median values of payments ranged from $366.8 to $9781.3 (Table 5). However, relatively small median payments were prevalent: 6 companies had medians lower than $1000. The IQRs ranged from $244.5 to $611.3 to $1834.0 to $48 906.7.

**Table 5.** Top 10 Donors^a^

Drug Company	Total Value, $ (%)	No. of Payments	Median (IQR), $
Pfizer, Inc	7 069 851.5 (9.8)	1774	427.9 (244.5-1834.0)
Bayer AG	5 383 697.1 (7.5)	2444	665.6 (332.0-1146.3)
GlaxoSmithKline	5 303 853.9 (7.4)	1224	366.8 (244.5-611.3)
AstraZeneca	4 780 547.8 (6.6)	1441	382.1 (267.5-764.2)
Novartis International AG	4 571 959.2 (6.3)	445	1222.7 (427.9-6113.3)
Biogen Idec	3 954 689.5 (5.5)	87	9781.3 (1834.0-48 906.7)
Janssen-Cilag GmbH	3 443 362.5 (4.8)	738	483.0 (302.6-1811.1)
UCB Pharma	3 235 289.0 (4.5)	64	2445.3 (616.0-11 128.1)
Hoffman-La Roche AG	2 514 572.5 (3.5)	171	1589.5 (611.3-6724.7)
Novo Nordisk A/S	2 022 117.3 (2.8)	346	468.3 (175.6-1467.2)

Abbreviation: IQR, interquartile range.

^a^ Payment values are expressed in US dollars. The 2015 annual average exchange rate from pounds sterling was used for the conversion (£1 = $1.53).

## Discussion

### Concentration of Payments

Financial relationships with the industry spread across the health care system, involving HCOs from different sectors (eg, public or private) and functions (eg, service provision or commissioning). However, consistent with the nature of the UK single-payer system, public-sector HCOs received the most funding, far outpacing third- and private-sector organizations. Separately, health care providers were the top recipients, which is unsurprising given their direct involvement in prescribing pharmaceuticals. The nonresearch funding received by HCOs involved in research and education and representing professional interests is notable, suggesting the industry sought to build positive relationships with organizations contributing to the development of new medicines or shaping the rules of their delivery. The extent of concentration within the recipient categories seemed higher than in the United States; although the GIs for 12 UK HCO categories ranged from 0.65 to 0.92, the respective range for 12 US medical specialties was 0.08 to 0.83.^37^ However, in both countries, differences in the concentration levels appeared to reflect varying sectors and functions, including a similar contrast between primary care (low concentration) and secondary care (high concentration).^37^

Payments concentrated on select payment categories, primarily donations and grants. However, the funding structure was different than that of payments to individual health care professionals,^10^ reported separately in Disclosure UK.^5^ Specifically, the only 2 payment categories reported for health care professionals—contributions to costs of events and fees for service and consultancy—constituted only 43.6% of HCO funding. This finding suggests that analyses of payments to health care professionals may miss other important types of industry financial ties to the health care system.^6,7^ Funding was also concentrated within payment categories, with a few large payments constituting the majority of the overall value.^38^ Joint working was the exception. The more equal distribution of payments in this category could reflect the unique nature of joint working, involving contributions of resources also from organizations participating in collaborative projects with the industry.

Funding from certain payment categories concentrated on some HCO categories. Contributions to costs of events accounted for the greatest share of funding of professional organizations, corresponding notably with the industry funding of medical society conferences.^39,40^ Donations and grants were prevalent with public health care providers, education and research providers, and charities. Because funding associated with this payment category excludes research expenditure,^10^ it corresponds with evidence of industry charitable donations and grants.^41,42,43^ Joint working was an important form of funding of health care commissioning and regulatory bodies, suggesting that collaborative industry-NHS projects were established at the strategic level in the health care system.^38^

Similar to payments to health care professionals,^5^ industry funding was concentrated by a few large companies. The concentration of funding might result from varying HCOs’ openness to accepting industry funding,^24^ company market access strategies, or resources available for payments. The consequences of payment concentration need further examination given the link, demonstrated in the United States, between receiving industry funding by health care professionals and increased brand name or costly prescription.^44,45,46,47,48^

### Patterns of Payments

Our results suggest a differentiation of industry funding strategies according to recipient and payment types. Some HCO categories, notably education and research providers and public-sector secondary and tertiary care providers, received larger funding per HCO than the other categories, possibly indicating their larger size and the resource-intensive nature of their activities. These HCO categories also displayed a sharper contrast between small and big beneficiaries of funding. In addition, a marked difference between public-sector secondary and tertiary care providers (a large number of smaller payments, translating into high amounts per HCO) and public-sector primary care providers (higher individual payments dispersed across a larger number of HCOs, resulting in lower amounts per HCO) was found. This difference might indicate that secondary and tertiary care HCOs (eg, hospitals) are larger and therefore more likely to have more frequent financial relationships with the industry than primary care HCOs (eg, local surgeries).

Similarly, the payment categories displayed distinct value patterns. Contributions to cost of events were a small-scale, low-intensity form of engagement with HCOs, whereas joint working payments suggested high-stakes projects, with a sharp contrast between those attracting a low and high amount of funding. Further, the similar pattern of payments for donations and grants and fees for service and consultancy suggests that these payment categories had a similar nature.

Notwithstanding the differences between recipient and payment categories, the top 10 donors shared an emphasis on making small payments, indicated by the broadly similar and relatively low median payment values. The importance of small payments corresponds with findings from the United States demonstrating their importance in building relationships with health care professionals.^15^

### Policy Recommendations

As with research on payments to health care professionals,^6,7^ our analysis of HCOs reveals data transparency shortcomings affecting Disclosure UK, which seem more serious than those associated with the Open Payments program.^14,49,50,51^ Notably, the Centers for Medicare & Medicaid Services have more robust mechanisms for monitoring and addressing inconsistencies in company payment data.^14^ Following the strengthening of regulations prohibiting companies from nondisclosure of payments to HCOs,^8,52^ the ABPI might use our categorization to develop straightforward HCO categories, thus enabling synchronic and diachronic analyses of payments across the health care system. As in the Open Payments program,^14^ unique identifiers would enable calculating the number and value of payments per HCO and the total number of HCOs. Similarly, adding information on products linked with payments would allow for analyzing relationships between industry funding and marketing strategies^53^ and clinical practice.^54^ Further, the full disclosure of payments for research and development would reveal industry engagement with HCO research activities.

Given the shortcomings of Disclosure UK, which is not compulsory for non-ABPI members and does not include medical device manufacturers, HCOs might consider developing their own disclosure systems. Given the discrepancies identified between payments reported by the industry and NHS organizations,^24,25^ the NHS might consider a central register of all external payments.

Related to the US context, our findings suggest that the $615.62 million paid to 1117 teaching hospitals in 2015 is probably a fraction of drug and medical device companies’ financial relationships with the health care system.^55^ Therefore, the inclusion of medical education and communication companies in the Open Payments program is a welcome step,^15,56^ and we believe it should be followed by other HCO categories, including education and research providers and professional organizations.

### Limitations

Our study has important limitations. First, given the challenges in preparing the data for analysis, we only cover the first year of Disclosure UK. However, the stability in the distribution of payments to health care professionals suggests that the same was the case for HCOs.^5^ Second, despite covering a vast share of the UK pharmaceutical field, the study is not exhaustive because non-ABPI members report payments voluntarily. Similarly, Disclosure UK does not contain information on the recipients of research payments. Third, we did not analyze payments to undisclosed recipients (2.9% of the total number). Fourth, we analyzed payments with a discrepancy between HCO categories in names and locations (10.4% of payments included in the analysis). Fifth, we disregarded possible differences between HCOs from different parts of the United Kingdom that might result from regional policies pursued in England, Scotland, Wales, and Northern Ireland.^57^ Sixth, unlike payments to patient organizations (subject to a separate disclosure system), HCO payments lack descriptions.^10,58,59^ Consequently, the exact purpose of activities covered by the ABPI’s 4 broad payment categories was not analyzed. Seventh, unlike in earlier analyses, we considered VAT to compare payment values reliably^6,7^; however, we most likely underestimated the value of payments by companies with no or unclear policy on VAT reporting.

## Conclusions

To our knowledge, this study offers the first comprehensive national-level analysis of pharmaceutical industry funding of HCOs in the United Kingdom or elsewhere. Despite being spread across the health care sector, industry funding concentrated on relatively few major donors, beneficiaries, and payments. The distinct patterns of payment values suggest a differentiation of funding strategies according to recipient and payment types. This study’s results suggest a need for the strengthening of industry- and government-run payment disclosure systems.

## Notes

Supplement. **eBox 1.** Disclosure UK Categories of Payments (Transfers of Value) to Health Care Organizations **eBox 2.** Definition of Health Care Organization According to the European Federation of the Pharmaceutical Industry and the Association of the British Pharmaceutical Industry **eFigure 1.** Lorenz Curve for All HCOs **eFigure 2.** Lorenz Curve for All Payments **eFigure 3.** Lorenz Curve for Drug Companies Based on the Value of Payments Made **eTable 1.** Categorization Framework for HCOs **eTable 2.** Drug Company Approaches to VAT Reporting **eTable 3.** Top 10 Recipients **eMethods.** Details of Data Collection and Management **eReferences.**
