# Supplementary material for: Analysis of Pharmaceutical Industry Payments to UK Health Care Organizations in 2015
Source: JAMA Netw Open. 2019 Jun 21;2(6):e196253. doi: 10.1001/jamanetworkopen.2019.6253 (PMC6593961; doi:10.1001/jamanetworkopen.2019.6253)
Supplement: Supplement. — eBox 1. Disclosure UK Categories of Payments (Transfers of Value) to Health Care Organizations eBox 2. Definition of Health Care Organization According to the European Federation of the Pharmaceutical Industry and the Association of the British Pharmaceutical Industry eFigure 1. Lorenz Curve for All HCOs eFigure 2. Lorenz Curve for All Payments eFigure 3. Lorenz Curve for Drug Companies Based on the Value of Payments Made eTable 1. Categorization Framework for HCOs eTable 2. Drug Company Approaches to VAT Reporting eTable 3. Top 10 Recipients eMethods. Details of Data Collection and Management eReferences. [file jamanetwopen-2-e196253-s001.pdf]

Supplementary Online Content

Ozieranski P, Csanadi M, Rickard E, Tchilingirian J, Mulinari S. Analysis of pharmaceutical industry payments to UK health care organizations in 2015. *JAMA Netw Open*. 2019;2(6):e196253.
doi:10.1001/jamanetworkopen.2019.6253

eBox 1. Disclosure UK Categories of Payments (Transfers of Value) to Health Care Organizations
eBox 2. Definition of Health Care Organization According to the European Federation of the Pharmaceutical Industry and the Association of the British Pharmaceutical Industry
eFigure 1. Lorenz Curve for All HCOs
eFigure 2. Lorenz Curve for All Payments
eFigure 3. Lorenz Curve for Drug Companies Based on the Value of Payments Made
eTable 1. Categorization Framework for HCOs
eTable 2. Drug Company Approaches to VAT Reporting
eTable 3. Top 10 Recipients
eMethods. Details of Data Collection and Management
eReferences.

This supplementary material has been provided by the authors to give readers additional information about their work.

© 2019 Ozieranski P et al. *JAMA Network Open*.

eBox 1. Disclosure UK Categories of Payments (Transfers of Value) to Health Care Organizations

1. "contributions towards the costs of meetings paid to healthcare organizations or to third parties managing events on their behalf
2. donations, grants and benefits in kind provided to institutions, organizations and associations
3. fees and expenses paid to healthcare organizations due to consultancy
4. contracts between companies and institutions, organizations and associations under which such institutions, organizations or associations provide any type of services on behalf of companies
5. joint working"

These payment categories **exclude** payments for Research and Development, defined as being "related to the planning and conduct of

- non-clinical studies (as defined in the OECD Principles of Good Laboratory Practice)
- clinical trials (as defined in Directive 2001/20/EC)
- non-interventional studies that are prospective in nature and involve the collection of data from, or on behalf of, individual or groups of health professionals specifically for the study".

Payments for research and development are reported "on an aggregate basis", that is as one lump sum per company, without disclosing their specific recipients.

(ABPI Code of Practice for the Pharmaceutical Industry)

© 2019 Ozieranski P et al. *JAMA Network Open*.

eBox 2. Definition of Health Care Organization According to the European Federation of the Pharmaceutical Industry and the Association of the British Pharmaceutical Industry

“Any legal person (i) that is a healthcare, medical or scientific association or organisation (irrespective of the legal or organisational form) such as a hospital, clinic, foundation, university or other teaching institution or learned society (except for patient organisations within the scope of the EFPIA PO Code[EFPIA Patient Organisation Code of Practice]) whose business address, place of incorporation or primary place of operation is in Europe or (ii) through which one or more HCPs provide services.” (EFPIA Code on Disclosure of Transfers of Value to Healthcare Professionals and Organisations and ABPI Code of Practice for the Pharmaceutical Industry)

© 2019 Ozieranski P et al. *JAMA Network Open*.

eFigure 1. Lorenz Curve for All HCOs

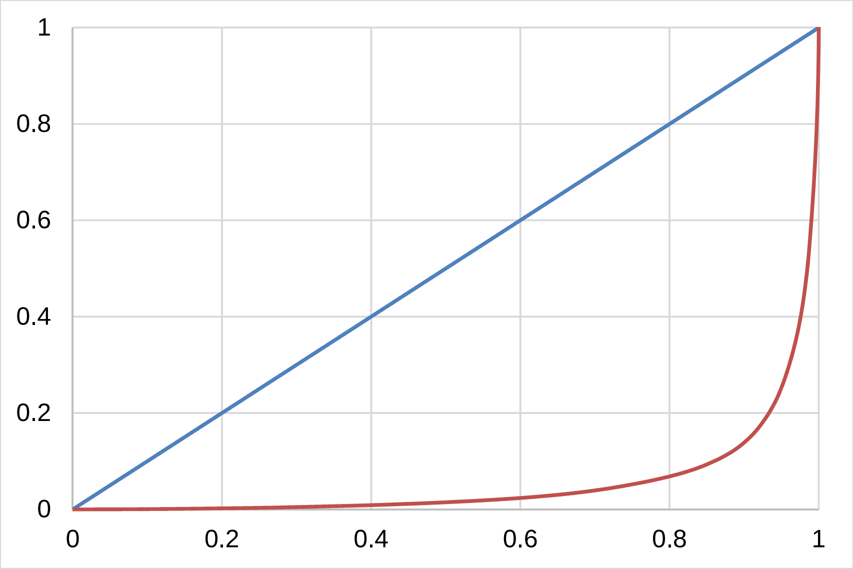

Abbreviations: HCO, healthcare organization

© 2019 Ozieranski P et al. *JAMA Network Open*.

eFigure 2. Lorenz Curve for All Payments

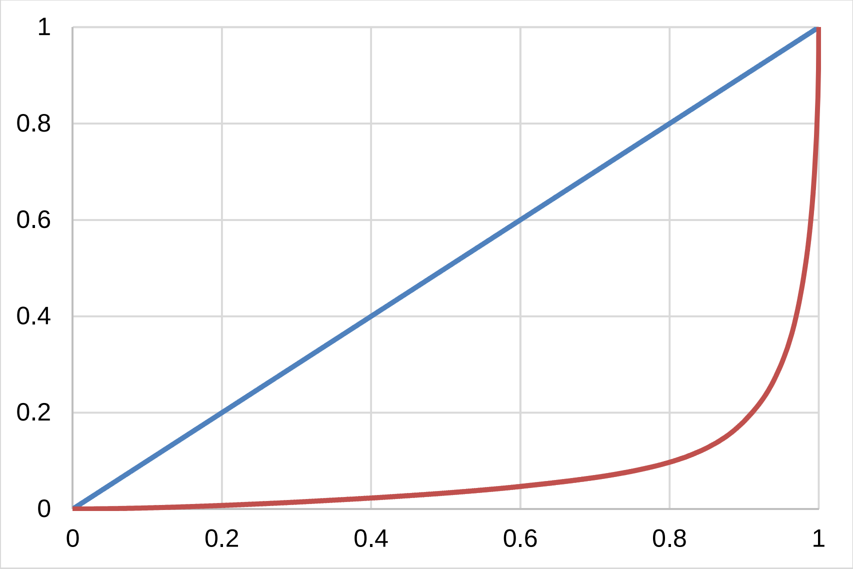

© 2019 Ozieranski P et al. *JAMA Network Open*.

eFigure 3. Lorenz Curve for Drug Companies Based on the Value of Payments Made

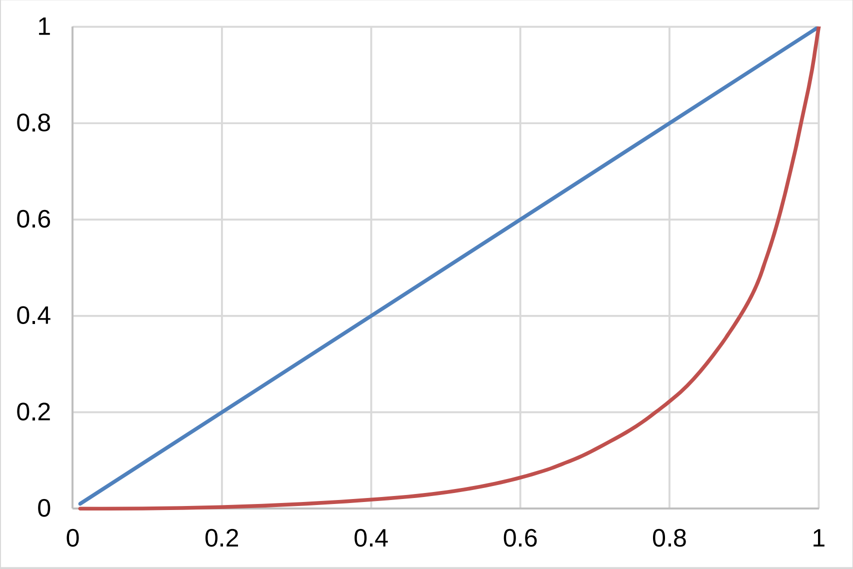

© 2019 Ozieranski P et al. *JAMA Network Open*.

eTable 1. Categorization Framework for HCOs

Legend

<u>Top-level HCO categories</u>

General HCO categories

Detailed HCO categories

**<u>Alternative providers of health services</u>**		
	**Associations of alternative providers of health services**	
		association of health and community health providers from private and voluntary sectors
	**Charities providing health services**	
		charitable organization providing community health or social care services
		hospice with a charitable status
		hospital with a charitable status
		nursing or care home with a charitable status
	**Not for profit companies providing health services**	
		not for profit company delivering community health or social care services
	**Social enterprises and community interest companies providing health services**	
		community interest company delivering health services
		social enterprise delivering health services
**<u>Organizations supporting patients, healthcare professionals or other organizations</u>**		
	**Multipurpose organizations**	
		multipurpose charity (e.g. fundraising, education, advocacy, patient support) but not a membership organization
		Multipurpose third-sector organization (non-charity)
	**Organizations focused on advocacy**	
		third-sector organization (non-charity) focused on advocacy
		charity focused on advocacy
	**Organizations focused on education**	
		third-sector organization (non-charity) focusing on education
		charity focused on education
	**Organizations focused on providing material support for patients or healthcare organizations**	
		third-sector organization (non-charity) focused on providing funding or material support to patients or NHS organizations
		charity focused on providing funding or material support to patients or NHS organizations
	**Organizations focused on providing patient support**	
		third-sector organization (non-charity) focusing on providing patient support
		charity focused on providing patient support (membership organization)
	**Organizations focused on research**	
		third-sector organization (non-charity) focused on funding medical research
		charity focused on funding medical research
	**Organizations with unclear purpose**	
		organization - unclear purpose
**<u>Education and research providers</u>**		
	**Other non-commercial research organizations**	

© 2019 Ozieranski P et al. *JAMA Network Open*.

charity focusing on undertaking medical research

non-university and noncommercial research institute funded by charitable organizations or public bodies

research institute, laboratory or registry - other

Universities

university

Formal bodies representing healthcare professionals or patients

Formal bodies representing healthcare professionals

local medical committees (LMC)

local optical or optometric committee (LOC)

local pharmaceutical committee (LPC) (England)

Formal bodies representing patients

organizations representing service users

Public healthcare commissioning, planning and regulatory organizations

Local and regional commissioning, planning or regulatory organizations

area pharmaceutical committee (APC) (Scotland)

area prescribing committee (APC) (England)

clinical commissioning group (CCG) England

collaboration of local and regional planning and provider organizations (England)

collaboration of regional NHS boards (Scotland)

commissioning support unit (CSU) (England)

health board (Wales)

local commissioning group (LCG) (Northern Ireland)

local commissioning group (LCG) (England)

locality group (England)

primary care trust (PCT)

regional NHS board (Scotland)

National-level commissioning, planning or regulatory organizations

collaboration of regional health boards - planning specialist services at the national level (Wales)

collaboration of NHS planning, commissioning and provider organizations (UK-wide)

Health and Social Care Board (Northern Ireland)

Health Education England (HEE)

MHRA

National Institute for Health Research (NIHR)

NHS Blood and Transplant

NHS England and bodies funded by NHS England

NHS National Services Scotland

NHS Shared Business Services

NICE

Public Health England

Public Health Wales

special NHS board (Scotland)

Private companies other than providers of health services

Industry trade groups or associations

industry trade group or association

© 2019 Ozieranski P et al. *JAMA Network Open*.

Private companies - unclear profile

"Educational support activities"

"general medical practice activities"

"hospital activities"

"Other business support service activities not elsewhere classified"

"Other education activities"

"Other human health activities"

"Other service activities not elsewhere classified"

"specialist medical practice activities"

company - unknown status

Providers of accountancy or consulting services

consulting services, including accountancy (excludes medical training and education but includes general training consultancy services)

Providers of commercial medical research services

clinical or contract research organization

private laboratory

Providers of medical communications or training services

company - discussion forum

event management services

journal or publishing company

medical communications (if consultancy services are provided in addition to communication then the company is coded as a consultancy; broader range of services than medical education and training; broader than event management;)

medical training or education services (if consultancy services are provided in addition to training/education then the company is coded as a consultancy)

Providers of medical technologies or services

facility services for hospitals

manufacturer or supplier of medical devices or technologies

pharmacy wholesaler or distributor

providers of information technologies

Retail companies

retail company

<u>Private sector healthcare providers</u>

Other private providers of health services

private company providing other health services e.g. nursing home

Private clinics and hospitals

private clinic, surgery or practice

private enterprise providing secondary or tertiary care treatments to NHS patients

private hospital

subsidiary within a NHS trust providing private patient services

Private healthcare groups

private healthcare group - primary, secondary or tertiary care

Providers of dental, pharmacy and optical services

dental practice

opticians

pharmacy or chemist

<u>Professional organizations</u>

© 2019 Ozieranski P et al. *JAMA Network Open*.

Multi-professional or multi-stakeholder organizations

alliance or coalition of professional associations or groups

multiprofessional organization - different healthcare professionals (e.g. medical professionals and nurses)

multiprofessional organization - healthcare and other professionals (e.g. doctors, nurses and scientists)

professional organization whose members are organizations

Organizations of medical professionals

organization of medical professionals (doctors) (more than one specialty)

organization of medical professionals (doctors) (single specialty)

Royal college - medical professionals

Organizations of other healthcare professionals

nurses' professional organization

professional organization of healthcare professionals other than medical professionals (doctors), nurses, pharmacists or pharmacy technicians

professional organization of pharmacists or pharmacy technicians

Royal college - healthcare professionals other than medical professionals or nurses

Royal college - nursing

Other professional organizations (non-healthcare professionals)

non-healthcare professional organizations (healthcare professionals not mentioned as a membership category)

Professional organizations with unclear membership status

professional organizations with unclear membership status

Public administration and providers of public services

Central UK government bodies

central government bodies

Crown dependencies

administrative bodies in UK crown dependencies

Devolved administrations

devolved administrations

Local authorities

borough council

city council

council - unitary authority

county council

district council

Prisons

prison

Public sector primary care providers

Federations and networks of primary care provider organizations

collaboratives of primary care organizations

federation, alliance, association, federation or consortium of GP practices

Primary care provider organizations

GP surgeries and health centers

group of surgeries or medical practices

healthcare or medical group

Public sector secondary and tertiary care providers

© 2019 Ozieranski P et al. *JAMA Network Open*.

Hospitals

NHS hospital

Networks and collaboratives of NHS organizations

academic health science network (AHSN)

clinical research network (CRN)

collaboratives or networks of secondary or tertiary care NHS organizations - disease specific

collaboratives or networks of secondary or tertiary care NHS organizations (including non-NHS stakeholders such as patients) - focus on research

managed clinical network (MCN) - Scotland

operational delivery network (ODN)

partnership between NHS organizations and other providers of health services - e.g. charities

strategic clinical network (SCN)

NHS trusts

NHS Foundation Trust

NHS trust

Recipients unclear

Recipients unclear

Unclear

Abbreviations: HCO, healthcare organization; NHS, National Health Service.

© 2019 Ozieranski P et al. *JAMA Network Open*.

eTable 2. Drug Company Approaches to VAT Reporting

Exclusive of VAT (net)
AbbVie Limited
Accretio
Actelion Pharmaceuticals UK Ltd
Alexion Pharma UK Ltd
Alimera Sciences Limited
Alliance Pharmaceuticals Ltd
Amgen Ltd
Astellas Pharma Ltd
AstraZeneca
Bausch & Lomb UK Ltd
Baxalta UK Ltd
Bayer Plc
BioMarin Europe Ltd
Consilient Health Ltd
CSL Behring
Daiichi Sankyo UK Ltd
Diurnal
Eli Lilly & Company Ltd
Ferring Pharma
Martindale Pharma
Merz Pharma UK Ltd
Napp Pharmaceuticals Ltd
Orion Pharma (UK) Ltd
Pharma Mar SA
Profile Pharma Ltd
Sandoz Ltd
Shionogi Limited
Sigma tau Rare Disease Ltd
STD Pharmaceutical Products Ltd
Sunovion Pharmaceuticals Europe Ltd
Thea Pharmaceuticals Ltd
Tillotts Pharma UK Ltd
**inclusive of VAT - gross**
A. Menarini Farmaceutica Internazionale S.r.l.
Actavis
Aegerion Pharmaceuticals Limited
ALK-Abello Ltd
Allergan Ltd
Almirall Ltd
Amdipharm Mercury Company Ltd
ApoPharma Inc

© 2019 Ozieranski P et al. *JAMA Network Open*.

Besins Healthcare (UK) Ltd
BGP Products Ltd
Bio Products Laboratory Ltd
Biotest UK
Bracco UK Ltd
Dermal
Eisai Ltd
Flynn Pharma
Gedeon Richter (UK) Ltd
Genzyme
Gilead
Grunenthal Ltd
Ipsen Developments Ltd
Lundbeck Ltd
Meda Pharma
Merck Serono Ltd
Novo Nordisk Limited
Octapharma Ltd
Orphan Europe UK Ltd
Sanofi Aventis
Sanofi Pasteur MSD
Servier Laboratories Ltd
Stirling Anglian Pharmaceuticals Ltd
Takeda UK Ltd
UCB Pharma Ltd
Vifor Fresenius Medical Care Renal Pharma
Vifor Pharma UK Ltd

no single rule

Alcon UK Ltd
Baxter Healthcare Ltd
Biogen Idec Ltd
Boehringer Ingelheim Ltd
Bristol-Myers Squibb Pharmaceuticals Ltd
Celgene Ltd
Chiesi Ltd
Chugai Pharma UK
Fresenius Medical Care (UK) Ltd
Galen Ltd
GlaxoSmithKline plc
Hospira UK Limited
HRA Pharma UK & Ireland Ltd
Janssen-Cilag Ltd
Leo Pharma Laboratories Ltd
Merck Sharp & Dohme Ltd
Norgine

© 2019 Ozieranski P et al. *JAMA Network Open*.

Novartis Pharmaceuticals UK Ltd
Otsuka Pharmaceuticals UK Ltd
Pfizer Ltd
Pierre Fabre Ltd
PTC Therapeutics Limited
Roche Products Limited
Santen UK Limited
Shire Pharmaceuticals Ltd
Sobi Ltd
Teva UK Limited

policy on VAT not mentioned

Britannia Pharmaceuticals
Guerbet Laboratories Ltd
Jazz Pharma
Mitsubishi Tanabe Pharma Europe Ltd
RB
Syner-med

© 2019 Ozieranski P et al. *JAMA Network Open*.

eTable 3. Top 10 Recipients[a,b]

Recipient name	HCO category	Total value $ (%)	Number of payments
King's College London	Education and research providers	3,931,007.2 (5.5%)	45
University of London - London School Hygiene and Tropical Medicine	Education and research providers	1,429,032.0 (2.0%)	16
PeerVoice	Private companies other than providers of health services	1,421,393.9 (2.0%)	11
University of London - University College London	Education and research providers	1,386,590.8 (1.9%)	96
Healthcare At Home	Private sector healthcare providers	1,333,839.4 (1.8%)	18
Central Manchester University Hospitals NHS Foundation Trust	Public sector secondary and tertiary care providers	1,130,349.7 (1.6%)	108
Quintiles - Scotland	Private companies other than providers of health services	1,043,243.2 (1.4%)	5
British Society for Rheumatology	Professional organizations	829,904.2 (1.2%)	31
King's College Hospital NHS Foundation Trust	Public sector secondary and tertiary care providers	780,916.2 (1.1%)	99
NHS Greater Glasgow and Clyde	Healthcare commissioning, planning and regulatory organizations	738,727.9 (1.0%)	153

Abbreviations: HCO, healthcare organization; NHS, National Health Service.
[a] Payment values are expressed in USD. The 2015 annual average exchange rate of USD / GBP was used for the conversion (£1=$1.53)
[b] See eTable 1 for the full framework and eTable 2 for the list of categories matching payments from Disclosure UK

© 2019 Ozieranski P et al. *JAMA Network Open*.

eMethods. Details of Data Collection and Management

This eAppendix provides the full list of steps taken in preparing Disclosure UK data at the HCO level for analysis for the purposes of this study.

The eAppendix follows the structure of the Methods section of the paper.

Data source – Disclosure UK

General information

Version of Disclosure UK

We analyzed the Disclosure UK dataset version 20160630, published on the 1st July 2016, and downloaded from the ABPI website on the same day.[1] We note the version and the dates because the have noticed that ABPI sometimes introduces minor changes to the dataset following its initial publication. The extent and nature of these changes are difficult to establish. The versions of Disclosure UK released in subsequent years have a different format so all statements made in this eAppendix refer to this particular version of Disclosure UK.

Identification of Healthcare Organizations in Disclosure UK

As the 2015 version of Disclosure UK does not have a separate column allowing for distinguishing between payments made to Healthcare Professionals (HCPs) and Healthcare Organizations HCOs, we extracted payments (otherwise termed Transfers of Value) to HCOs by filtering out rows in the dataset with no values entered in the "First Name" and "Last name" columns, which are filled for payments made to HCPs but not HCOs. We assumed that the lack of entry in those columns indicated that the payment recipient was a HCO.

Variables in Disclosure UK associated with HCOs

There are 13 variables applicable to HCOs in the version of Disclosure UK we analyzed: Company Name, Organization Name, Location, City, Country of Principal Practice, Address Line 1, Address Line 2, Post Code, County, payment Category, paymentType, payment amount and Joint Working Link.

© 2019 Ozieranski P et al. *JAMA Network Open.*

There are four payment categories: Contribution to costs of Events, Donations and Grants to HCOs, Fee for service and consultancy, and Joint working

Contribution to costs of events include three more detailed payment types: Registration Fees, Sponsorship agreements with HCOs, and Travel and Accommodation.

Fee for service and consultancy include two more detailed payment types: Fees and Related expenses agreed in the fee for services or consultancy contract.

Donations and Grants to HCOs and Joint working appear in both paymentcategories and payment types.

For the purposes of our study, we rely on payment categories rather than on payment types as they have a smaller number of more comprehensive values.

Cleaning payment values

Before proceeding to analysis, we turned 20 payments with negative values, worth $160,210.2, into positive ones. We assumed that the negative values resulted from the lack of standardisation of accounting systems between and within companies.

Categorizing Healthcare Organizations

Three levels of categorization

Our categorization framework has three primary levels.

- The **detailed level** captures unique characteristics of payment recipients, including the regional differences between HCOs based in England, Scotland, Wales and Northern Ireland.
- The **general level** aggregates the characteristics of detailed categories, disregarding, in particular, differences between England, Scotland, Wales and Northern Ireland, and focusing on HCO features that are shared across the UK.

© 2019 Ozieranski P et al. *JAMA Network Open*.

- The **top-level** includes the most general HCO characteristics that could be found in any Western health system. This level of the categorization is most suitable for international comparisons.

Supplementary principles of categorization

We introduced **supplementary principles of categorization** so as to

- distinguish organizations from the third sector from organizations from the private, public and mixed (public and third) sectors
- distinguish between different categories of organizations from the third sector (i.e. Alternative healthcare providers, Organizations supporting patients, healthcare professionals or other organizations, or Professional organizations)
- distinguish between different types of organizations included in each top-level category of organizations from the third sector

There were two additional principles of categorization

- **Charitable status** – indicated by the registration with the charity regulator in England and Wales (Charity Commission for England and Wales[2]), Scotland (Scottish Charity Regulator[3]) or Northern Ireland (Charity Commission for Northern Ireland[4]). To establish the membership status of organizations we copied and pasted their names into the online search engines of the three charity regulators and checked whether they had any records associated with their names. We categorized an organization as having the charitable status if it was registered with at least one of the three charity regulators.
- **Membership status** - indicated by the characteristics of members of HCOs (e.g. one or more than one medical specialty) as provided on the Membership sections of their websites (and if not available, on their main websites).

© 2019 Ozieranski P et al. *JAMA Network Open.*

Development of the categorization framework for healthcare organizations and its validity

We developed the categorization framework for HCOs iteratively. This process involved the following steps.

- We started by coding all payment recipients using detailed categories. Subsequently, we aggregated the detailed categories to create general and top-level categories. We were refining the category names at all levels throughout the categorization process. This involved merging or splitting existing categories as well as developing new ones. These revisions were intended to allow for organizations to be grouped together more adequately. We also sought to reduce the number of codes, especially at the detailed level, to maximize the clarity of the presentation of findings.
- Each level of categorisation was applied in relation to the recipient of each payment. The presence of three levels, including the detailed one, was crucial for ensuring the validity of decisions about placing payment recipients in specific categories. The categories associated with each payment recipient can be scrutinised in the Online Supplement linked with this publication.

Website checks

Sources of data

We extracted data from the healthcare organizations' main webpage, and when necessary, the "About us", "History" or "Membership" sections. If an organizational website was not available, we used other available websites mentioning the organization to minimize the number of payments with unclear recipients. In particular, we checked the following websites.

- The Companies House website[5] – to establish whether a healthcare organization was a private company

© 2019 Ozieranski P et al. *JAMA Network Open.*

- The websites of the Charity Commission for England and Wales, Scottish Charity Regulator and the Charity Commission for Northern Ireland to establish whether a healthcare organization was a registered charity.

Dealing with discrepancies between Disclosure UK and website data

In rare instances of discrepancies between recipient names reported in Disclosure UK and on the organizational websites we relied on the information provided on the websites when assigning the organization categories to payment recipients. For example, if a health center (Disclosure UK) appeared as a medical practice during the web search it was coded as medical practice. All instances of differences between Disclosure UK and the websites were minor and affected only the detailed level of coding.

Timing of web searches

The timing of the web searches (February-May 2017) was close to the time when the payments were made (until the end of 2015, with the database released in June 2016). Therefore, it is reasonable to assume that the results of the web searches provided an accurate capture of the nature of healthcare organizations.

With very few exceptions, the healthcare providers listed in Disclosure UK still existed at the time of the searches. However, some private sector companies providing health services and private companies other than providers of health services had ceased to exist. In a vast majority of these cases we were able to establish their status as closely as possible to 2015 based on the Companies House Website.

Inter-coder reliability

Two researchers, ER and PO, coded half of the dataset each at the detailed level, with any revisions being discussed and adopted in real time. PO then coded the payments using the general and top-level categories and checked the consistency of coding between and within categories at detailed, general and top-level. The consistency checks focused on ensuring that

- organizations covered by the same code were of the same nature;

© 2019 Ozieranski P et al. *JAMA Network Open*.

- organizations covered by different codes were sufficiently different;
- each organization was assigned one category at the detailed, general and top-level.

Distinguishing between different sectors of healthcare provider organizations

When deciding about the sector of healthcare providers (public, private, third) we applied the following principles:

- **Public sector secondary and tertiary care providers –** we assumed that all hospitals were NHS hospitals (that is, public sector hospitals) unless it was specifically stated on their main website that they were private healthcare organizations or alternative healthcare providers (e.g. social enterprises, community interest companies or charities). However, if a ward or unit within an NHS trust provided services exclusively for private patients we categorized it as a private sector healthcare provider.
- **Public sector primary care providers –** we assumed that all primary care organizations (e.g. surgeries, health centers, clinics) were public sector if they were listed on the NHS website or had an NHS logo on their website. We did not investigate separately whether they provided additional health services for private patients.
- **Alternative healthcare providers –** we assumed that a healthcare organization was an alternative healthcare provider if its main website specifically mentioned that it was a community interest company, social enterprise, or a charity.

Naming Healthcare Organizations

Notation of "Names" and "Locations"

We use the terms "Names" and "Locations" in inverted commas when referring to the way in which they were used in Disclosure UK. We do not use inverted commas when referring to the approach to naming HCOs established for the purposes of this study.

© 2019 Ozieranski P et al. *JAMA Network Open*.

Approach to reporting HCO names taken in this study

As noted in the paper, we report our results by HCO names and categories based on "Locations" unless they were categorized as unclear; in that case they were substituted by "Names". There were three reasons. First, "Locations" had fewer unclear recipients (1,987, 9.9%) than "Names" (2,940, 14.7%). Second, using "Locations" minimized the loss of information associated with arriving at a single level of aggregation for the HCO category receiving the greatest number and value of payments, that is secondary and tertiary public healthcare providers (see Results). Specifically, both "Names" and "Locations" comprised a combination of NHS trust and hospital names, but the share of the former was considerably greater in "Locations". It was therefore straightforward to replace NHS hospitals in "Locations" by their respective trusts, and not the other way round (NHS trusts typically comprise more than one hospital). Third, focusing on the trust level allowed for reducing the complexity of the dataset and comparing the number of HCOs with other main UK healthcare statistics (e.g. NHS Confederation data on NHS trusts).

The HCO categories in "Names" and "Locations" disagreed in relation to 2,093 (10.4%) payments. These payments were spread across different donors, HCO and payment categories. We kept them in the analysis as the general rule of prioritizing information from "Locations" allowed for resolving these discrepancies; for example, we interpret 130 payments to primary and 980 to secondary care organizations ("Names") as having been made to healthcare commissioning organizations ("Locations") which fund their activity.

Addressing inconsistencies in HCO names reported in Disclosure UK

In creating the list of HCO names reported here, we addressed inconsistencies in the naming of HCOs in Disclosure UK, such as the same HCO being referred to with different names (e.g. "NHS Bristol CCG [Clinical Commissioning Group]" and "Bristol CCG") and different HCOs appearing under the same name (e.g. Grove House Surgery in West Yorkshire and Somerset).

First, we standardized the original HCO "Names" (4,224, 21.1% entries changed) and "Locations" (7,550, 37.7%) by removing typos and introducing consistent spelling. Second, we simplified the

© 2019 Ozieranski P et al. *JAMA Network Open*.

standardized HCO “Names” (1,167, 5.8% changes) and “Locations” (1,237, 6.2%) to allow for reporting the same HCOs at a single level of aggregation (for example, at the NHS trust level and not the trust, hospital, or department levels for different payments). Third, we placed the simplified HCOs from “Names” (208, 1.0% changes) and “Locations” (481, 2.4%) at the same level of aggregation particularly by replacing hospitals from “Names” with their respective trusts from “Locations”. Fourth, we generated the final list of HCO names by replacing unclear “Locations” by the “Names” associated with the same payments. Finally, we used postcode information to eliminate any remaining instances of different organizations not being counted separately or the same HCOs appearing under different names.

Website checks

Consistent with the evolving nature of the healthcare system, the names of some of healthcare organizations changed between the time when the payments were made and the time of the web checks. For example, some commissioning or regulatory organizations had merged or been renamed (e.g. two clinical commissioning groups became one clinical commissioning group). Similarly, some NHS trusts merged or ceased to exist.

In these instances, we did not change the organization names reported in Disclosure UK as in some instances this would affect the number of healthcare organizations in the dataset.

© 2019 Ozieranski P et al. *JAMA Network Open*.

eReferences

- 1. ABPI. Explore the data further. 2019.
- 2. Charity Commission for England and Wales. Find a charity. 2019; http://apps.charitycommission.gov.uk/showcharity/registerofcharities/RegisterHomePage.aspx.
- 3. Scottish Charity Regulator. Search. 2019; https://www.oscr.org.uk/search.
- 4. Charity Commission for Northern Ireland. Charity search. 2019.
- 5. Companies House. Search the register. 2019; https://beta.companieshouse.gov.uk/.

© 2019 Ozieranski P et al. *JAMA Network Open*.
